# Equipoise across the patient population: optimising recruitment to a randomised controlled trial

**DOI:** 10.1186/s13063-016-1711-8

**Published:** 2017-03-27

**Authors:** Paul Whybrow, Robert Pickard, Susan Hrisos, Tim Rapley

**Affiliations:** 1 Newcastle University, Baddiley-Clark Building, Newcastle Upon Tyne, NE2 4AX UK; 2 Institute of Cellular Medicine, The Medical School, Newcastle University, 3rd Floor William Leech Building, Newcastle upon Tyne, NE2 4HH UK; 3 Present address: School for Social and Community Medicine, Canynge Hall, 39 Whatley Road, Bristol, BS8 2PS UK

**Keywords:** Trials, Recruitment, Qualitative, Multicentre, Trial design, Surgical trial, Equipoise, Patient preferences

## Abstract

**Background:**

This paper proposes a novel perspective on the value of qualitative research for improving trial design and optimising recruitment. We report findings from a qualitative study set within the OPEN trial, a surgical randomised controlled trial (RCT) comparing two interventions for recurrent bulbar urethral stricture, a common cause of urinary problems in men.

**Methods:**

Interviews were conducted with men meeting trial eligibility criteria (*n* = 19) to explore reasons for accepting or declining participation and with operating urologists (*n* = 15) to explore trial acceptability.

**Results:**

Patients expressed various preferences and understood these in the context of relative severity and tolerability of their symptoms. Accounts suggest a common trajectory of worsening symptoms with a particular window within which either treatment arm would be considered acceptable. Interviews with clinician recruiters found that uncertainty varied between general and specialist sites, which reflect clinicians’ relative exposure to different proportions of the patient population.

**Conclusion:**

Recruitment post referral, at specialist sites, was challenging due to patient (and clinician) expectations. Trial design, particularly where there are fixed points for recruitment along the care pathway, can enable or constrain the possibilities for effective accrual depending on how it aligns with the optimum point of patient equipoise. Qualitative recruitment investigations, often focussed on information provision and patient engagement, may also look to better understand the target patient population in order to optimise the point at which patients are approached.

**Trial registration:**

ISRCTN Registry, ISRCTN98009168. Registered on 29 November 2012.

## Background

About a half of all randomised controlled trials (RCTs) face difficulties in achieving target recruitment [1]. Recruitment shortfall has ethical implications and comes at considerable cost in terms of time and effort to the patients and trialists, as well as wastage of patient data and monetary loss. Recruitment and retention are widely felt to be the most important factors for achieving a successful trial [2, 3]. Nesting a qualitative study within trials is seen as an effective method of improving recruitment [4] but there is a lack of methodological and theoretical articulation of how qualitative findings achieve this. This paper reports findings from a qualitative study embedded in the feasibility phase of the ‘Clarifying the management of men with recurrent urethral stricture: a pragmatic multicentre, randomised superiority trial of open urethroplasty versus endoscopic urethrotomy (OPEN)’ trial (www.opentrial.co.uk). The purpose of the study was to improve trial recruitment by investigating patient experience, clinicians’ perspectives and overall trial process.

### Qualitative research and trial recruitment

In the United Kingdom (UK) the National Institute of Health Research (NIHR) recently set a target to see more patients and health professionals participating in health research [5]. Trialists are challenged to reverse the trend of poor participation through improved trial design and management. Qualitative studies have emerged as key to addressing this challenge. A recent review found that trials with a qualitative component were better equipped to adapt to problems and increase recruitment [4]. Embedded qualitative investigations are described as the ‘most successful intervention’ to address poor recruitment ([4]: 12). However, these studies can vary in quality and depth and are often poorly reported [6]. Successful implementation of qualitative findings requires that the research is integrated within the trial, carried out by experienced qualitative researchers and develops robust conceptual articulation of the data [7].

Donovan et al. have demonstrated the value of analysing clinician and patient trial-orientated interactions, focussing on recruitment activity, to improve patient information and the recruitment process [8]. The way that clinicians present the interventions, the need for randomisation and study rationale has a significant impact on whether eligible patients feel that participation is acceptable [9]. Trial recruitment is a fragile process as clinicians may experience discomfort where their instinct may be at odds with the community equipoise and require training and support to adjust [10]. Qualitative research can be used to expose barriers hindering recruiters from engaging productively with eligible patients [11, 12].

The most significant barrier to trial recruitment is patient preference for a specific treatment option [13–16]. A number of studies have explored why patients accept or decline trial participation, revealing factors such as forms of (conditional) altruism, a sense of involvement or therapeutic misunderstandings [17, 18]. In practice, patients’ preferences are complex and often trial-specific [6, 19] so it is important that they are acknowledged by recruiters and brought into an information exchange [9, 12, 20].

As well as paying attention to clinician and patient perspectives, it is also important to look at the congruence of trial design and standard care pathways [21, 22]. Practical and contextual considerations often become highly important to trial success such as needing to proactively follow up participants [21]. Qualitative research can have an evaluative role in identifying issues with the trial design or poor enactment [23]. Treweek has pointed out that trials often succeed or fail on the basis of early design decisions and calls for a better awareness of practical design choices [21, 24].

The nature of qualitative research is situated and context-driven, which means that its distinct value can be difficult to pin down. Advocates refer to the ability to ‘explore complexity’, ‘identify barriers’ and understand ‘social contexts’ [4, 8], although it is difficult to provide definitive guidelines. Trialists should be mindful of the inability to explore all areas in a short period of time, and focus on aspects most important to their trial [7]. In this paper we build on these discussions using the findings from our qualitative investigation of recruitment to the OPEN trial, demonstrating how they informed changes to the trial recruitment strategy. We suggest that there is benefit in better understanding the patient population and the conditions in which patients are in equipoise.

### Equipoise and trial participation

The concept of equipoise is the ethical foundation for recruiting patients into clinical trials. It has emerged from the legal-moral discussions about the conditions under which it is ethically permissible to randomise a patient’s treatment as part of a clinical trial [25], the concern being that recruiting to a trial may compromise the professional duty of care for individual patients. It is argued that such a responsibility is not undermined where none of the treatments are felt to be a superior option for the patient. In other words there is neither advantage nor disadvantage to the patient in either selecting a treatment or having their treatment randomly allocated [26, 27]:

‘Equipoise is the point where we are equally poised in our beliefs between the benefits and disadvantages of a certain treatment modality. … At this point we are agnostic or resting on the fulcrum of a preference.’ (Johnson et al. 1991: p. 30 in [28])

‘Individual equipoise’ (or ‘theoretical equipoise’) is where an individual clinician is balanced in their opinion of the available treatments. ‘Collective equipoise’ (or ‘community equipoise’) is where there is no consensus among the profession as a whole [25, 27–29]. Collective equipoise is the basis from which individual clinicians are often urged to put aside personal opinions and accept the collective uncertainty of their peers in order to recruit to a trial [27, 29].

Equipoise is often discussed in terms of adequate or sufficient uncertainty; however, it is important not to confuse this with a lack of knowledge [30]. There is often extensive evidence as to the safety and effectiveness of trial arms. The key aspect of equipoise is that, given the current evidence, there is an equilibrium between the alternatives [28]. Establishing this uncertainty in practice can be very difficult. Consider, for example, that most contemporary trials do not involve like-for-like outcomes, such as the eradication of a cancer, but multiple outcomes, such as quality of life measures or recovery time. Lilford and Jackson helpfully distinguish ‘absolute equipoise’, in which a treatment is either better or worse, with the more common ‘effective equipoise’, in which there exists a trade-off between multiple outcome measures [29]. Many contemporary trials, like the OPEN trial discussed in this article, involve effective equipoise in which the balance between treatments involves a trade-off between known differences in the treatments.

Within trial literature the concept of equipoise has been discussed almost exclusively from the perspective of professionals and the medical community (the ‘we’ in the Johnson et al. quote above, is referring to professionals). The literature on shared decision-making and equipoise tends to focus on how best to communicate clinical or collective equipoise to a patient, rather than on whether patients themselves are in equipoise [10, 27, 30]. However, the idea of being fully informed yet poised between alternative treatments is entirely applicable to patients’ decision-making. Ethical trial recruitment requires that patients are informed yet have no preference between treatments [31]. The prominence of clinical rather than patient equipoise in the trial literature is a reflection of how the field developed within highly clinical, often oncological, trials which presuppose a particular form of patient-professional relationship, where the professional has specialist knowledge and the patient is required to accept equipoise by proxy [31]:

‘In theory a patient who gives informed and voluntary consent to enter a randomised trial has achieved the equilibrium of equipoise. In practice equipoise among patients ranges from personal to proxy.’ (Alderson, 1996: p. 135 in [31]).

However, there is no need for equipoise by proxy where the necessary information is easily communicated and understood by patients, as they would be able to reach personal equipoise themselves. With regards to recruitment, trialists often work on the basis that patients are most amenable to participation when they adequately comprehend clinical equipoise. What has not yet been explored, to the best of our knowledge, is how patients’ equipoise may encompass value-driven factors that differ from the evidence-based clinical uncertainties of the trial. Neither has it been considered that the patient population, like the professional community, may have collective equipoise. In this paper we use the concept of equipoise to understand patients’ decisions about whether or not to participate in the trial.

### The OPEN trial

Bulbar urethral stricture is a narrowing of the urethra caused by scar tissue and is a common cause of urinary difficulties in men. The OPEN trial compares the effectiveness of two surgical interventions: urethrotomy and urethroplasty. Urethrotomy is a relatively straightforward operation in which the scar is incised using an endoscope passed down the urethra. However, the scar tissue and urinary difficulties often recur typically within 2 years. Urethroplasty is a more invasive operation which involves reconstructing the urethral tube through a skin incision between the legs and using graft tissue taken from inside the mouth. The OPEN trial has established urological centres across the UK to randomise men with recurrent urethral stricture between these two surgical interventions. The eligibility criteria is to be 16 years or older with no upper age limit. Men must have a bulbar urethral stricture and have undergone at least one previous intervention for the stricture. However, there are no exclusion criteria for the severity of symptoms.

Accrual for the OPEN trial faces particular challenges that require investigation. Participants must be willing to have their intervention allocated at random. Furthermore, the treatment options differ in invasiveness and the duration of postoperative bladder catheterisation. The relatively low prevalence of recurrent urethral stricture entails the need for multiple recruitment sites across the UK, leading to variation in recruitment practices and resources as well as clinician bias [1]. Lastly, men with the condition often prefer to self-manage and conceal symptoms rather than seek curative treatment [32]. To explore these challenges, the OPEN trial employed a nested qualitative study, interviewing both patients and clinicians to better understand and inform feasibility of the recruitment process.

## Methods

Interviews were with 19 men who were eligible for participation in the OPEN trial and 15 urologists seeing and treating men with urethral stricture. Patients were approached about trial and qualitative study participation during their treatment decision-making at urology sites across the UK. At the close of the qualitative component, 25% (40/159) of those screened for trial participation said that they would be willing to take part in an interview. To be eligible for the trial, a patient will have had one previous intervention for urethral stricture; all those interviewed had, therefore, undergone previous stricture surgery but their symptoms had returned. The researcher (PW) contacted all those who were willing to be interviewed. Following ethics approval (reference: 12/NE/0343) and individual informed consent, semistructured interviews explored men’s experience of the disease, their treatment preferences and their experience of trial recruitment. Of the 19 patients interviewed, 9 men had also consented to treatment randomisation and 10 had declined full trial participation.

Fifteen clinicians were also recruited from a list of practicing urologists in the UK and included some who were recruiting to the trial (*n* = 9) and others who were either not involved (*n* = 2) or who were considering participation (*n* = 6). The researcher (PW) contacted clinicians by telephone or email and conducted the audio-recorded interviews either face-to-face or by telephone. Recordings were transcribed verbatim, anonymised, then coded and analysed following the broad principles of thematic analysis [33, 34]. Analysis was undertaken alongside data collection, so analysis of prior interviews informed both the sampling decisions and interview questions. We used Qualitative Data Analysis (QDA) software to support the management and retrieval of data [35], as well as writing analytic memos and using tables, process maps, and diagrams to further explore and refine emergent issues [34]. Analysis was also supported by discussion of anonymised transcripts in trial meetings and regular qualitative data clinic sessions, which included health professionals and social scientists from a range of clinical and academic backgrounds.

## Results

The findings are reported in three sections: *Patient preference*, *Clinicians’ perspectives* and *Organisation of care and trial recruitment.* The purpose is to illustrate how the qualitative investigation improved our understanding of patient preference and clinician practice, and how these could be mapped to the existing trial design.

### Patient preference

Nineteen men, ranging in age between 25 and 70 years old (median = 36), were recruited from five urological clinics. Most of the men interviewed had had one or two previous urethrotomies, three had had three or more repeat urethrotomies. Bulbar urethral stricture is a benign urological condition that significantly impacts on men’s lives. The main symptoms are frequent and prolonged visits to the toilet that mean waking during the night, disruption to work and social life, and the threat of social embarrassment [32]. The patients interviewed were sympathetic to the purpose of the trial, although, as with other trials, often declined participation because of an overriding preference for one option.

#### Preference for urethrotomy

Those men who expressed a preference for urethrotomy said that it was to avoid wearing a catheter, taking time off work or undergoing the ‘serious’ operation necessary for urethroplasty:

‘I didn’t fancy losing 3 months, 4 months off work, couldn’t afford to.’ (Patient, declined)

‘You’re going to be in hospital for a couple of days you have the catheter in for over 10 days or what not. It’s kind of, no it’s freaked me out a little bit. I’m quite happy with urethrotomy.*’* (Patient, declined)

The shorter recovery period and minimum disruption to their work and lifestyle appealed to them and could be underpinned by the private nature of the condition. Concealment, particular to conditions such as bulbar urethral stricture, reinforces the tendency to avoid time off work or wearing a catheter [32].

Patients were aware of the trade-off between repeated treatments versus the possibility of a permanent solution:

‘Nobody really fancies surgery but [the clinician] says I should have [urethroplasty] done and I said, “well I will next time and I will make the time for it”. I’ll have to because if you add up all the time I’ve had for [urethrotomy] that I’ve lost I could have been sorted by now.’ (Patient, declined)

Bulbar urethral stricture, although a significant burden on men’s lives, is a benign condition that lacks the sense of fear and immediacy that other patients might experience with conditions such as cancer. This means that these men could reasonably opt for the ‘short-term solution’ of urethrotomy and potentially delay curative treatments:

‘The operation I’ve already had, I’m quite happy with that until it’s really necessary to move on.’ (Patient, declined)

Delaying a more serious operation (urethroplasty) until ‘moving on’ is necessary or unavoidable, was a common justification for having a preference for urethrotomy:

‘I don’t think I could go through that operation unless anything drastic happened [yeah] where I really feel, you know, the pain was getting too much.’ (Patient, declined)

#### Preference for urethroplasty

Other interviewees declined randomisation due to an overriding preference for urethroplasty. The common account of these patients is that they are seeking a curative solution because the symptoms and recurrence, despite previous urethrotomy, were no longer tolerable. For these patients being randomised to another urethrotomy was not acceptable:

‘I’ve tried that and it didn’t work, why would I do it again?’ (Patient, declined)

Some of these men who were actively seeking an alternative to urethrotomy, had been referred for that purpose and had travelled some distance to see a urethroplasty specialist. These patients saw themselves as being at a point in which urethrotomy was not an acceptable treatment option. As the following extract illustrates, they can be understood as being past the point of equipoise:

‘I don’t see the sense in sitting here in 6 months’ time going in for regular dilation because, you know, there has to come an end to it somewhere. So that’s the route I went down. And I said [to the consultant], “look you know, I’m very happy to help in any way that I can [with the trial] but, eh, I’d rather go for the sort of bigger op on the basis that there’s a good chance that will sort it out and make life more comfortable for a longer period of time”. And that’s where I’m at*.*’ (Patient, declined)

A key detail is the idea of being *at a specific point*, or as this participant said ‘that’s where I’m at’. This is representative of other accounts of declining trial participation due to a preference for urethroplasty. It should be noted that there was no particular number of previous urethrotomies that helped to frame the decision: some were unwilling to have a second, others were willing to receive a third or fourth.

#### Accepted randomisation

The nine men interviewed who accepted randomisation described being at a point in which the difference in cost and benefits of each treatment were negligible. Three expressed a weak preference but were still willing to ‘help out’. The other six said that, given the evidence, they were undecided which treatment would be best for them at that point in time. These patients can be understood as being at a point of equipoise as they are informed, having weighed the comparative costs and benefits of the two alternative treatments, yet have no strong preference. They are, with the current information they have to hand, agnostic, or equally poised, between the desire for a shorter recovery time and the possibility of a curative solution:

‘[With urethroplasty] I’m not so worried about the catheter. I would take the time off and just sit that out […] But it’s, obviously, [worrying] that you will make a full recovery afterwards and you won’t be having to look after your wounds forever, essentially. That’s the worry on that. [Consultant] got a very good reputation for this sort of thing. So it’s not this actual procedure that bothers me, it’s the recovery afterwards and affecting your ability in later life. […] As for concerns about the urethrotomy, my main concern with that is that I don’t want to keep having that every 2 years. It was a relatively painless operation. I was in and out during the same day. But I don’t want to keep going back every 2 years or so to have that repeatedly done.’ (Patient, randomised)

This patient decided to have his treatment randomised and is a good example of a point of equipoise within the OPEN trial. Having had two previous urethrotomies, he was willing to have another but also ready to try the more invasive alternative. The man describes being worried both about recovering from urethroplasty and needing repeat surgery, which are the uncertainties underpinning the OPEN trial. This balance of factors illustrates the necessary uncertainty for accepting random treatment allocation and trial participation. Interestingly, however, the same patient commented that, were there to be a next time, he would no longer be indifferent:

I: ‘Would you feel disappointed at all if you were randomised to the urethrotomy?’

R: ‘I’m not disappointed at this stage, no. I think, obviously, the next time, I would probably be heavily in favour of the other.’ (Patient, randomised)

Such accounts suggest a particular window of opportunity in which men with recurrent bulbar urethral stricture are in equipoise and willing to accept randomisation.

Patients’ balanced the immediate inconvenience of a long recovery period with better chances of a curative solution. Table 1 illustrates how treatment decisions were closely related to patients’ perception of the severity and manageability of their symptoms. Those with an overriding preference understood their own urethral stricture symptoms to be at a particular point: *either too slight to consider a serious operation* or *too severe not to*. An important contribution of the feasibility qualitative study is to highlight how equipoise happens within particular margins of symptom severity and manageability.

**Table 1:** Summary of patients’ accounts of their treat preferences

Decision	Preference	Symptoms	Operation
Declined randomisation	Preference for urethrotomy	Symptom recurrence and severity is tolerable	Symptom recurrence sufficiently tolerable to not want to endure serious operation and recovery time
	Preference for urethroplasty	Symptom recurrence and severity no longer tolerable. Patient desires a permanent solution	Desire for long-term solution overrides immediate symptom relief. Unwilling to risk further recurrence
Accepted randomisation	No preference	Symptoms tolerable but considering serious operation	Willing to commit to recovery time. An additional repeated urethrotomy is also acceptable

### Clinicians’ perspectives

The OPEN trial is multicentre with participating urology clinics across the UK. Fifteen clinicians from 15 sites were interviewed in order to better understand their perception of the trial and recruitment practices. Their years of experience range broadly from 4 to more than 30 years. Some clinicians reported common concerns around time constraints, resourcing and questions regarding patient eligibility. All clinicians were supportive of the OPEN trial and felt that it would answer a valuable clinical question. An important distinction to make at this juncture is that there are two types of clinicians participating in the trial: specialist urologists and general urologists. Specialists are able to offer both of the OPEN trial treatment options as they are trained to carry out urethroplasty, whereas general urologists are only able to deliver urethrotomy and would refer patients to a specialist if they feel that urethroplasty is required. A key finding, and purpose of this section, is that general and specialist urologists differed in their approach to recruitment.

#### General urologists

Most general urologists anticipated that eligible patients would prefer urethrotomy and that this would be a barrier to recruitment. General urologists’ accounts presented this as the ‘easy’ option that patients would ‘obviously’ prefer:

‘If you give the patient the option of having an endoscopic procedure where he goes home the same day versus being referred to another unit somewhere else for an open surgical procedure then obviously patients might go for the easier option.’ (General urologist)

‘Most patients are going to opt for a urethrotomy and not a urethroplasty but you can either have day case operation and go home with a catheter for a day or two or you can have a major procedure and be in hospital for a few days and I think it’s just going to be hard: why would I want to be randomised, I’d rather just have the easy one.’ (General urologist)

These expectations were related to ideas about the average types of eligible patient. General urologists described men with recurrent stricture as being older or with relatively minor symptoms. These men were typically treated by repeated urethrotomies and self-management using the adjunctive technique of intermittent self-dilatation (ISD). Putting these patients forward for randomisation would be challenging:

‘I think the work [of the OPEN trial] needs doing, it’s a good study to do. I think we will have a few issues with people opting to have an operation that we may not have recommended in the normal situation.’ (General urologist)

Although eligible, randomising these patients proved difficult when at odds with their routine clinical practice. The following quote suggests a blurred line between what the ‘old guys’ want and what the general urologist is comfortable offering:

‘There are few guys, *old* guys, who really don’t want to have an urethroplasty that keep getting urethrotomy. You know most of them, after I have seen them, will go with the urethrotomy and then send him home generally.’ (General urologist)

The perceived expectations of the ‘older patients’ or those with ‘relatively minor symptoms’ underlie the clinician’s temptation to be selective in identifying potential participants:

‘If somebody has a very simple urethral stricture and then you will think “oh, why should I subject this person to an open procedure rather than just an optical urethrotomy?”’ (General urologist)

‘I think we do treat the different age groups slightly differently so the young guys are more likely to go for a urethroplasty […] whereas your elderly guy, you’re trying to avoid operations, […] I think there is a lot of individual basis that we are going to make these decisions on.’ (General urologist)

Uneven representation of the treatment arms can be seen to be an extension of what the clinician felt that their patients wanted or needed. The perceived expectation of the ‘typical’ patient with recurrent stricture led to a tendency to recommend urethrotomy:

‘Patients who would like to take the chance [with urethroplasty] if you tell them that with the urethrotomy there is a certain chance that you’ll be fine you won’t need the reconstruction so they might go for that’. (General urologist)

The findings of the qualitative study, therefore, highlight a preference, a selection bias, of general urologists (See Table 2). However, it is important to note how this preference is underpinned by consideration of patients who tends to be older or with relatively minor symptoms.

**Table 2:** Comparison of trialists’ expectations and recruitment practices

Trialist	Patient population	Clinicians’ expectations	Recruitment practice
General urologist	Older, history of self-management and urethrotomy	Patients will opt for urethrotomy	Older patients excluded, reluctance to refer
Specialist urologist	Younger, complex referrals	Patients will opt for urethroplasty	Reluctance to suggest a repeat urethrotomy

#### Specialist urological surgeons

Specialist clinicians anticipated the opposite: that few patients would be willing to consider another urethrotomy and that overriding preference for urethroplasty would be a barrier to recruitment (see Table 2):

‘I would imagine for somebody who’s had two urethrotomies, of average – of typical age, probably 75% of them definitely are happy to proceed to the urethroplasty.’ (Specialist urological surgeon)

Specialists see a large proportion of referrals with either severe or complicated strictures referred specifically with the intention of discussing urethroplasty as a treatment option:

‘One of [my] concerns with the OPEN trial is that the guys I’m seeing are generally those who have been referred to me with a view to doing an urethroplasty.’ (Specialist urological surgeon)

‘They may have already had two or three urethrotomies and now they are referring them on with a view to a urethroplasty.’ (Specialist urological surgeon)

Specialists found it hard ‘selling’ the trial to patients and felt compelled to guide them towards urethroplasty, especially cases that were severe or complex. Here, a specialist describes an encounter with a man who has been tolerating severe symptoms:

‘He said, “oh that’s about *normal* (!)”. I said, “well if I told you that was less than 10% of the flow of a normal person and that you’re leaving behind more than half a bladder full of urine, how would you react to that?” And he said, “oh that’s terrible!” I said, “yes, well you really should have an [urethroplasty] operation!”’ (Specialist urological surgeon)

In this case, despite the patient’s eligibility and uncertainty, the surgeon could not remain neutral and was compelled to recommend urethroplasty. Such accounts are evidence of a lack of clinical equipoise at the limits of the eligibility criteria [10, 27]. Many specialists described the difficulty in staying neutral. Even if they would not explicitly recommend urethroplasty, they reported producing language and terminology representing treatment arms unevenly:

‘If you say to them you can have a more complicated operation you’ve got a 95% chance of being cured versus we can keep doing this [urethrotomy] every couple of years and you’ll end up urethral cripple, most of them will take the option [of urethroplasty].’ (Specialist urological surgeon)

The eligibility criteria for the OPEN trial has no upper age limit. Neither is the severity of the stricture symptoms a reason for exclusion from the study. However, interviews reveal how, in practice, this can be grounds for selectivity in approaching patients. Previous nested qualitative studies have highlighted clinician ‘selection bias’ as a distinct barrier to recruitment. Similarly, our result found that despite supporting the trial, general and specialist urological surgeons admitted to only discussing the trial with certain types of patient, or struggling to represent the arms of the trial evenly. These findings highlight the need for training to support and promote more standardised recruitment practices across participating sites [36]. However, the difference between general and specialists invites a closer look at how the trial is integrated with the organisation of care.

### Organisation of care and trial recruitment

The OPEN trial qualitative feasibility study found evidence that patient preference and clinician ‘selection bias’ are potential barriers to trial recruitment. As with previous trials, it is important to engage with these issues through such actions as improved patient information and recruiter training [36]. The findings also illustrate how patients may have a particular window in which they are in equipoise and that clinical selection bias reflects differences in general and specialist practice. In this section we map these findings onto the organisation of stricture patient care and trial recruitment design. Patients reported changes to their preferences and anticipated changing their preference in the future. This illustrates how preferences are dynamic and have a temporal dimension. Those who opted for urethrotomy felt that it was too soon to consider the alternative and those who opted for urethroplasty felt that it was too late not to. So although preference between individual stricture patients differed, there exists a common trajectory underpinning these decisions. Patients in equipoise described being at a particular point in which differences in cost and benefit of each operations were negligible.

Once these men seek help they will typically be referred by a general practitioner to a urological clinic. At this point a lot depends on geographical location; the majority will go to their nearest general urological clinic while others go to a specialist centre. In the first instance, urethral stricture patients are most likely to be offered a urethrotomy. Many of these patients will experience symptom recurrence anywhere between 3 months to several years and will return to the clinic to discuss further treatment. This is the point at which the patient is eligible take part in the OPEN trial.

Taken alongside clinicians’ accounts, this suggests that the population of eligible urethral stricture patients is unevenly represented between general and specialist sites. General urologists have a higher proportion of stricture patients receiving repeated urethrotomy treatments, either because their symptoms are relatively minor and tolerable, or because they wish to avoid a serious operation or travelling to a specialist. Specialists are more likely to encounter patients considering urethroplasty and who may have been referred for that purpose.

At the onset of the OPEN trial recruitment (spring 2013) all patients were being recruited from specialist centres. This made practical and organisational sense: consultants involved in setting up the trial were based at these sites and were able to deliver both treatment arms. However, the qualitative findings illustrate how patients approached at this point tended to be ready for urethroplasty and are, therefore, not in equipoise. Patients reported a common trajectory of worsening symptoms underpinning their decision about trial participation. What was needed was to recruit patients earlier in their treatment pathways, before specialist referral, when their symptoms are tolerable enough to consider a repeat urethrotomy. One way to do this, and part of the recommendations of the feasibility study, was to increase involvement of the general urology sites to capture patients earlier in their condition.

## Discussion

The aim of the OPEN trial is to establish the relative benefit and cost-benefit of urethroplasty and urethrotomy over 2 years for treating men with recurrent bulbar urethral stricture. Urethral strictures can be treated either with a urethrotomy, which is essentially a symptom palliation procedure, or the more time-consuming and invasive urethroplasty, which may be a curative solution. In order to participate in the OPEN trial, patients need to be equally poised in their belief about the relative benefits and disadvantages of each treatment.

### Timing of approach

**Fig. 1:**
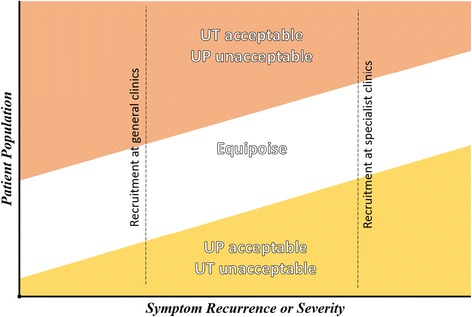
An epidemiological characteristic of equipoise: points of recruitment capture different proportions of the patient population

The results highlight how patients’ decisions to accept or decline randomisation were situated within the overall trajectory of their illness and treatment. Generally, the longer a patient has had a urethral stricture, the more likely it is that their symptoms will return and the more likely they are to want to commit the time necessary for the possible curative solution promised by urethroplasty (see Table 1). Patients who felt that their symptoms were tolerable were less likely to commit to the recovery time and more likely to opt for urethrotomy. Therefore, patient preference can be understood within the context of a stricture patient’s common trajectory of worsening symptoms. There was a particular window of opportunity in which patients were willing to accept either procedure. The OPEN trial initially relied on approaching patients at specialist sites. However, this was misaligned with the point at which the patients were most likely to be in equipoise (Fig. 1). Following the feasibility study, the OPEN trial has opened more general centres in order to capture patients earlier in their illness trajectory. This change to recruitment practice had the desired improvement to recruitment. General site recruited at an average of 30% of screened patients compared to 27% in the specialist centres. However, setting up the additional sites was resource-intensive for the trial team.

### Clinical and collective equipoise

The qualitative interviews with clinicians found, similarly to other trials, inconsistent recruitment practices, selectivity and conflicts between trial recruitment and local clinical practice. This was to be expected as recruiting to a clinical trial is known to be emotionally and intellectually challenging, especially where it conflicts with routine clinical judgements [10]. Recruiters often struggle to offer trial participation with patients on the limits of eligibility [10, 27], such as extreme ranges of age or symptom severity. A unique finding within this study is the difference in clinical bias between general and specialist sites. Specialist urologists were reluctant to randomise patients whose symptoms were severe, while general urologists found it difficult to refer patients’ whose symptoms were manageable with a repeat urethrotomy. These differing perspectives correspond with the types of stricture patient they typically treat. General urologists see patients with moderate symptoms while specialists see more complex and severe cases (see Table 2).

We suggest that the difficulty that recruiters have in achieving clinical equipoise is not adequately explained as individual bias, but is partially an outcome of their relative position in the organisation and division of urethral stricture care. As the eligibility criteria for the trial includes patients with moderate and severe symptoms, the target population is unevenly distributed between sites. As a whole, the urological profession achieves collective equipoise. However, individual recruiters may struggle to align their practice with clinical equipoise when they manage a particular proportion of the patient population (see Table 2).

These findings add to the evidence that recruiting to a clinical trial is highly challenging and the importance of training to help clinicians overcome individual bias and communicate the delicate issue of collective equipoise [22, 27, 36]. Furthermore, we suggest that the difficulties faced were differentiated and reflect the structural and organisational contexts of clinicians’ practice. Understanding these perspectives can help to develop effective and targeted training and other recruitment interventions.

### Patient population and equipoise

In this research we used both patients’ and clinicians’ accounts to understand how patients’ experiences map onto the point at which patients are approached about the trial. Patients’ decision about whether or not to participate in the OPEN trial were understood within an expected trajectory of worsening symptoms. Patients who declined participation either felt that their condition was too slight to consider a serious operation or too severe not to. Patients who agreed to randomisation described being at a particular point in time when the relative cost and benefits of each procedure where negligible.

The concept of equipoise, as informed uncertainty, can be applied to patients’ decision about trial participation. Those patients who were willing to be randomised were at a point in their condition where the advantages or disadvantages of recovery time or of repeat surgery were equivalent. The findings suggest that treatment preferences were dependent on how serious or intolerable the patients felt their condition to be. Although these factors are balanced from the perspective of the urological community, they may be considerably uneven for an individual patient who values recovery or a curative solution above all else. Patients declining trial participation were not just expressing a preference, they were making informed decisions based on how the known trade-off between the procedures relates to their relative condition. In this sense the point of patient equipoise can be understood within the context of the experiences of the broader population of stricture patients (Fig. 1).

While not all trials will involve patient equipoise related to a trajectory of worsening symptoms, they may involve patient equipoise relative to other factors of the target population such as gender [37, 38]*,* behavioural change [39] educational level [40] or patient involvement [41]. The specific nature of these factors is dependent on the particularities of the trial, the patient population and the interventions being compared. Qualitative research can be used to better understand a target patient population and reasons for patients having uncertainty between treatments, particularly where these depart from the conditions of clinical equipoise.

### Qualitative research and trial recruitment

A number of methods have been suggested to overcome the ‘problem’ of patient preference. Statistical techniques can adjust for patient preference [42] or clinician biases [43], although they risk confounding results [42]. Patient preference trials or recruiting from cohorts also sidestep the issue as these can only compliment rather than replace the ‘(gold) standard’ of randomisation [44]. Qualitative research embedded within trials has emerged as the most promising approach to improving recruitment [4] by, for example, successfully helping clinicians to better engage with patients [10]. Qualitative data requires careful application by development of explanatory accounts using sets of analytical techniques [45]. There has been a tendency to over-simplify qualitative research and reifying ‘patient preference’ into a linear ‘barrier’ to be overcome. For example, saying that patients’ self-completed questionnaires will ‘establish recruitment barriers’ by avoiding ‘interviewer bias’ [46], which implicitly assumes that patients are best placed to directly answer recruitment problems. Similarly, others refer to interviewing clinicians as problematic because it is an indirect way to gain access to patient opinions [47, 48]. These approaches are limited because they reduce the role of qualitative research to simply canvassing opinions. In contrast, interviewing clinicians may be crucial for understanding the range of patients typically seen and the context of invitations to participate. Interviewing patients is helpful in understanding their lived experience or reasons for decision-making. Such research works best when it is theoretically informed and used to develop explanatory accounts of that patient group.

In their work of reviewing and categorising qualitative research within trials, O’Cathain et al. found that trialists rarely had a good rationale for employing qualitative studies and urge that embedded qualitative methods should be more explicit and directed [7]. We hope to have contributed to this discussion, suggesting that one way in which to guide qualitative studies within trials is to overtly explore and elucidate key factors underpinning treatment preferences and equipoise. We recommend thinking about patient equipoise as potentially involving values external to those of the trial and how these may impact on the possibilities for recruitment.

## Conclusion

The findings of the OPEN trial qualitative study are comparable to those of other recruitment investigations in finding that *patient preference* and *clinician selection bias* are significant in limiting trial recruitment. The particular value of these findings for the OPEN trial has been in showing how and why these differ between site types. Stricture patients are unevenly represented between points of recruitment. Patients with the sufficient uncertainty to be in equipoise were approached at the appropriate point in their illness trajectory when neither treatment was felt to be superior. General and specialist clinicians had different expectations of patients and can be seen to struggle to align their practices with the collective equipoise of the trial. The implication of these findings for the OPEN trial was to recruit patients earlier in the referral process by enrolling more general urology sites, and to develop differentiated, targeted recruitment training for general and specialist urologists.

We have suggested that patient and clinician preference, often discussed as ‘barriers’ to recruitment, are often an artefact of the organisation of care and the point at which patients are approached. Qualitative feasibility studies can be used to better understand the target population prior to recruitment in order to optimise the point of approaching patients. The conditions of patient preference and equipoise are likely be contingent on values other than those of collective equipoise underpinning the trial. Articulating these findings requires an epidemiological approach to the concept of equipoise, situating it as a measurable characteristic of a target patient group.

## Abbreviations

ISD: Intermittent self-dilatation
NIHR: National Institute of Health Research
OPEN: Clarifying the management of men with recurrent urethral stricture: a pragmatic, multicentre randomised superiority trial of open urethroplasty versus endoscopic urethrotomy
QDA: Qualitative Data Analysis
RCT: Randomised controlled trial
UK: United Kingdom
